# Phenotyping of a new yeast mapping population reveals differences in the activation of the TORC1 signalling pathway between wild and domesticated yeast strains

**DOI:** 10.1186/s40659-024-00563-5

**Published:** 2024-11-07

**Authors:** Guilherme Rocha, Melissa Gómez, Camila Baeza, Francisco Salinas, Claudio Martínez, Eduardo I. Kessi-Pérez

**Affiliations:** 1 Centro de Estudios en Ciencia y Tecnología de Alimentos (CECTA), Universidad de Santiago de Chile (USACH), Santiago, Chile; 2 Departamento de Ciencia y Tecnología de los Alimentos, Universidad de Santiago de Chile (USACH), Santiago, Chile; 3 Laboratorio de Genómica Funcional, Instituto de Bioquímica y Microbiología, Facultad de Ciencias, Universidad Austral de Chile, Valdivia, Chile; 4 ANID-Millennium Science Initiative-Millennium Institute for Integrative Biology (iBio), Santiago, Chile

**Keywords:** Domestication, Natural variation, Nitrogen sources, *Saccharomyces cerevisiae*, TORC1 activation, Yeast, Wild yeasts, Wine yeasts

## Abstract

Domestication can be understood as a symbiotic relationship that benefits both domesticator and domesticated species, involving multiple genetic changes that configure the phenotype of the domesticated species. One of the most important domesticated species is the yeast *Saccharomyces cerevisiae*, with both domesticated strains used for different fermentations processes for thousands of years and wild strains existing only in environments without human intervention; however, little is known about the phenotypic effects associated with its domestication. In the present work, we studied the effect of domestication on yeast TORC1 activation, a pleiotropic signalling pathway conserved across the eukaryotic domain. To achieve this goal, we improved a previously generated methodology to assess TORC1 activation, which turned out to be as effective as the original one but also presents several practical advantages for its application (such as facilitating confirmation of transformants and putting the *Luc* reporter gene under the control of the same *P*_*RPL26A*_ promoter for each transformed strain). We then generated a mapping population, the so-called TOMAN-G population, derived from the “1002 Yeast Genomes Project” population, the most comprehensive catalogue of the genetic variation in yeasts. Finally, strains belonging to the TOMAN-G population were phenotyped for TORC1 activation, and then we compared the results obtained between yeast strains with different ecological origins, finding differences in TORC1 activation between wild and domesticated strains, particularly wine strains. These results are indicative of the effect of domestication on TORC1 activation, specifically that the different evolutionary trajectories of wild and domesticated strains have in fact caused differences in the activation of this pathway; furthermore, the phenotypic data obtained in this work could be used to continue underlying the genetic bases of TORC1 activation, a process that is still not fully understood, using techniques such as GWAS to search for specific genetic variants underlying the observed phenotypic variability and phylogenetic tree inferences to gain insight into the evolutionary relationships between these genetic variants.

**Supplementary Information:**

The online version contains supplementary material available at 10.1186/s40659-024-00563-5.

## Background

Domestication processes are important because they mark important transitions in human history [1]. They can be understood as symbiotic relationships that benefit both the domesticating and the domesticated species [2], involving multiple genetic changes that shape the phenotype of the latter [3]. Although many of the studies in this area focus on the domestication of animals and plants [4], domestication events are not limited to them; in fact, one of the most important domesticated species is the budding yeast *Saccharomyces cerevisiae*, due to its use for food and beverage production for thousands of years [4]. Importantly, *S. cerevisiae* presents both domesticated strains distributed in human-intervened environments and wild strains widely distributed in nature in habitats far from human activity [5, 6]. However, little is known about the phenotypic effects linked to domestication in yeast.

Furthermore, *S. cerevisiae* is considered a model organism for biological sciences, being the first eukaryotic organism with its genome fully sequenced in 1996 [7]. Since then, several attempts have been made to unravel the genetic diversity and population structure of the species. The first of them confirmed the presence of two yeast populations, domesticated yeasts and wild yeasts [8–10], while more recent works have expanded the number of subpopulations [5, 11–13]. In this context, the most exhaustive study of genetic variation in *S. cerevisiae* was the “1002 Yeast Genomes Project”, in which a highly diverse population of 1011 yeast strains was sequenced, thus including most of the genetic variation existing in the species [13].

One of the main domestication niches of *S. cerevisiae* is the wine fermentation environment. This yeast is considered the main microorganism responsible for the alcoholic fermentation in the winemaking process, contributing with the alcoholic degree, flavours, and aromas to the final product [14, 15]. Grape must is a challenging environment for yeasts, where it is necessary to cope with ethanol toxicity, low pH (between 2.5 and 3.5), high sulphite levels, high osmotic pressure (20% of sugar concentration), and, most importantly, limited nitrogen availability [16, 17]. Nitrogen deficiencies in grape juice impair yeast biomass production, and, therefore, fermentation rate, causing stuck or sluggish fermentations, which in turn generate economic losses for winemakers [18, 19]. Moreover, yeast growth rate is not only associated with the amount of available nitrogen, but also with the quality of the nitrogen source [20]. In the context of fermentation, nitrogen sources sustaining high growth rate are “preferred” (consumed first), while “non-preferred” nitrogen sources are consumed at later times during fermentation [21]. This preference for different nitrogen sources is the result of a tight regulation system, in which four different mechanisms regulate nitrogen utilization (known as SPS, NCR, RTG, and GAAC), and all of them are in turn regulated by the TORC1 signalling pathway [22, 23].

TORC1 corresponds to a pleiotropic signalling pathway, conserved across the eukaryotic domain, that connect nutrient availability with growth, playing a central role in general metabolism regulation, especially linked to nitrogen metabolism [24]. This pathway is inhibited by rapamycin [25, 26], with rapamycin-resistance experiments being the classic way to study TORC1 activation. In yeast, the presence of nitrogen sources activates the TORC1 complex, inducing processes such as protein biosynthesis, amino acid biosynthesis, translation initiation, and ribosome biogenesis; whereas nitrogen starvation conditions lead to the inhibition of the TORC1 complex activity, inducing autophagy, stress response genes, nitrogen catabolic genes, and ammonium permeases [27]. This explains the close relationship that exists between TORC1 activation and wine fermentation, especially under low nitrogen conditions [24, 28].

It has also been reported that this pathway could be rapidly but transiently activated by both “preferred” and “non-preferred” nitrogen sources, but a sustained activation is caused only by “preferred” nitrogen sources [29]; however, it is not fully understood how TORC1 is activated by these different nitrogen sources (*i.e.*, how nitrogen availability information is transmitted to the TORC1 complex) [22, 23, 30, 31]. And regarding domestication, it is important to note that there is evidence that indirectly suggests that domestication has caused differences in TORC1 activation between wild and domesticated yeast strains [32–35]. However, the low number of strains evaluated so far do not allow to determine whether there is a general trend towards the existence of differences of TORC1 activation between different yeast strains due to the domestication process.

In the present work, we generated a mapping population, the so-called “TOMAN-G” population (for **TO**RC1 activation **Ma**pping of **N**ew variants by **G**WAS), derived from the “1002 Yeast Genomes Project” population, composed of 274 diploid-euploid yeast strains with a high genotypic and phenotypic diversity. We then took advantage of a recently developed method for indirect monitoring of TORC1 activation in microculture, which uses luminescence as a readout of pathway activation by nitrogen sources, allowing to phenotype a large number of strains [35]. Our results show that there are differences in TORC1 activation between wild and domesticated strains, particularly wine strains, suggesting that the domestication process has caused differences in the activation of this signalling pathway. This phenotypic information could be useful to study the genetic determinants underlying TORC1 activation by experimental approaches such as QTL (Quantitative Trait Loci) mapping or GWAS (Genome-Wide Association Studies), and therefore to study the distribution of different alleles between wild and domesticated yeast strains and the evolutionary history of genes associated to TORC1 activation by phylogenetic tree inferences [36, 37].

## Results

### Improvement of a method to assess TORC1 activation

To study the effect of domestication on TORC1 activation, we selected the “1002 Yeast Genomes Project” population, a population of 1011 yeast strains with wild and domesticated origins [13]. In order to phenotype this large population, we needed a high-throughput and fast-to-implement methodology. Therefore, we took advantage of a method for indirect monitoring of TORC1 activation in microculture recently developed in our laboratory, which uses luminescence as a readout of pathway activation, allowing phenotyping a large number of strains [35]. This method considered the transformation of the yeast strains to be evaluated with a reporter construct containing the firefly luciferase gene (*Luc*), designed to replace the endogenous ORF of the *RPL26A* gene by homologous recombination, so that luciferase expression would remain under the control of the endogenous *RPL26A* promoter (*P*_*RPL26A*_) of each transformed strain [35].

**Fig. 1:**
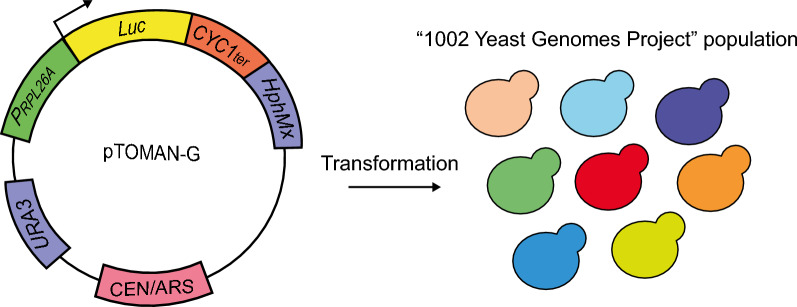
Construction of the pTOMAN-G plasmid. The main features for the expression and replication of the plasmid in yeast are shown. The *RPL26A* promoter (*P*_*RPL26A*_), a downstream target of TORC1, was used to control the expression of the luciferase (*Luc*) reporter gene. The genetic construct was assembled in the centromeric pRS316 plasmid, including the *CYC1* terminator (*CYC1*_*ter*_) and the hygromycin cassette (*HphMx*) as a selectable marker. It also kept the original *URA3* gene from the pRS316 plasmid as another selectable marker. This plasmid was used to transform a subpopulation of diploid-euploid strains derived from the “1002 Yeast Genomes Project” population

While this methodology proved useful for phenotyping a recombinant biparental population composed of 96 yeast strains [28], several concerns were raised about its use to study a larger, more genetically diverse population, including the laborious task of confirming transformants by PCR, the selectable marker used, and the fact that the reporter gene is always under the control of a different promoter (*i.e.*, the endogenous *P*_*RPL26A*_ promoter of each transformed strain). To avoid these problems, we designed and constructed a single-copy plasmid carrying the reporter gene (*Luc*) (Fig. 1). We called this plasmid the “pTOMAN-G” plasmid, for **TO**RC1 activation **Ma**pping of **N**ew variants by **G**WAS, since in addition to the study of TORC1 activation in domesticated and wild yeast strains, another goal of the present study was to generate a mapping population for genotype–phenotype correlation techniques like GWAS (or, alternatively, QTL mapping). This plasmid has two key features: (i) it has an antibiotic resistance gene (*HphMx*) as a selectable marker, while also maintaining the *URA3* gene for auxotrophic laboratory strains, which facilitates the confirmation of transformants; and (ii) the *Luc* gene is now under the control of the *P*_*RPL26A*_ promoter of the BY4741 laboratory strain, being the same for each strain transformed with this plasmid. In sum, this new plasmid allows a simpler way to transform yeast strains, while maintaining the rest of the experimental protocol for assessing TORC1 activation as was previously published [35].

### Generation of the TOMAN-G population

**Fig. 2:**
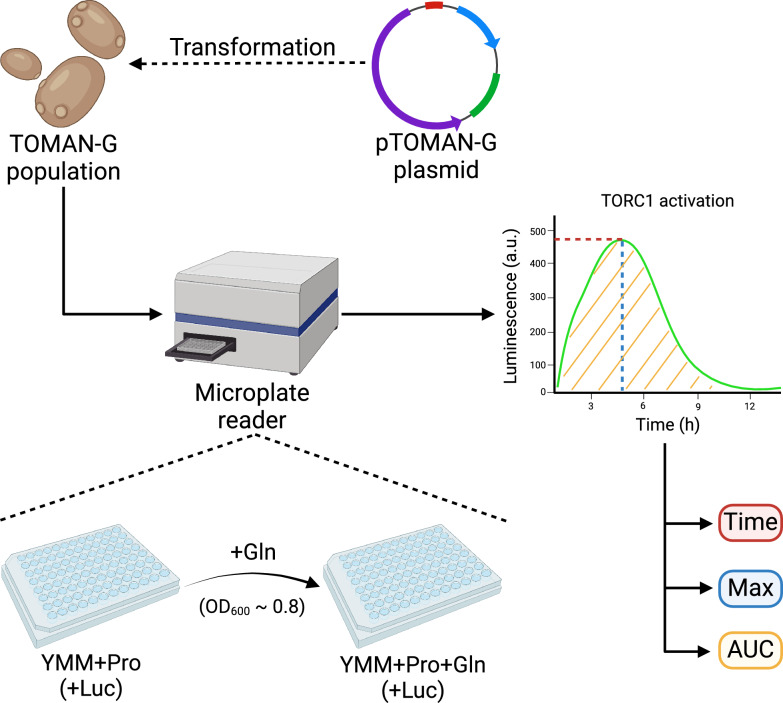
Overview of the method for indirect monitoring of TORC1 activation using the TOMAN-G population. The TOMAN-G population was generated by transforming 274 yeast strains that were originally part of the “1002 Yeast Genomes Project” population with the pTOMAN-G plasmid. These strains were evaluated in nitrogen upshift experiments by growing them in 96-well plates with YMM + Pro medium and adding glutamine (Gln) as a nitrogen source when the strains reached OD_600_ ~ 0.8. The luminescence (Lum) was recorded after addition of glutamine every 10 min. [Created in BioRender.com]

An important requirement for yeast strains to be phenotyped with the selected method is their ability to grow on proline as the sole nitrogen source, reaching at least an optical density at 600 nm (OD_600_) of 0.8 (Fig. 2), a common requirement for different methods that are based on performing nitrogen upshifts from a “non-preferred” nitrogen source (such as proline) to a “preferred” one (such as glutamine) [28, 29, 35, 38, 39]. As the “1002 Yeast Genomes Project” population is highly diverse both genetically and phenotypically, we first studied the ability of the strains belonging to this population to grow on YMM + Pro medium in microculture. We paid especial attention to diploid-euploid strains, as strains with aneuploidies and/or different ploidies (haploidy or polyploidy) can have large confounding effects when included in GWAS [40]. We also categorized each yeast strain in the population as “wild”, “domesticated (wine)”, “domesticated (non-wine)”, or “unknown”, following previous criteria [40, 41].

**Fig. 3:**
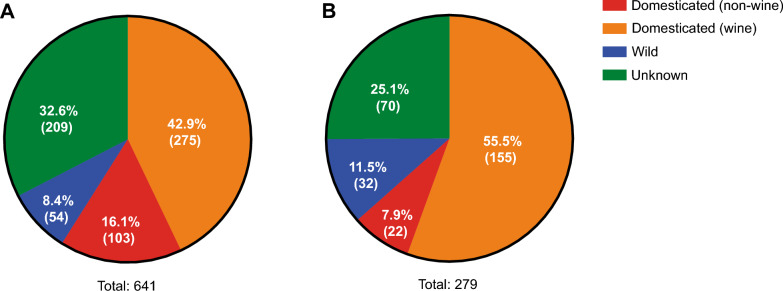
Class composition of the different populations under study. The yeast strains in each group are those **A** diploid-euploid belonging to the “1002 Yeast Genomes Project” population and **B** capable of growing in the YMM + Pro medium

Of the 641 diploid-euploid strains evaluated, only 279 (43.5%) were able to grow to at least OD_600_ = 0.8 in 46 h, the time limit we imposed for this evaluation (Fig. 3). Interestingly, strains with wild and domesticated (wine) origins retained a higher representation (59.3% and 56.4%, respectively) than strains with domesticated (non-wine) or unknown origins (21.4% and 33.5%, respectively) (Fig. 3), which greatly changed the composition of this population compared to the 641 diploid-euploid strains. It should be noted that the composition of the 641 diploid-euploid strains was also different compared to the total of 1011 strains, but to a lesser extent; the subset of 641 diploid-euploid strains showed a higher representation of domesticated strains (both wine and non-wine) than the original population, but the percentage differences were smaller than those previously mentioned (Fig. 3 and Additional file 2: Figure S1). However, even with these compositional changes, of the 26 clades and 3 mosaic groups originally proposed in the “1002 Yeast Genomes Project” [13], 24 clades and all 3 mosaic groups retained at least one representative strain (Additional file 1: Table S2), thus not losing much genetic diversity compared to the original population.

We then took these 279 strains and transformed them with the pTOMAN-G plasmid. Since they are very genetically diverse, we used two different transformation methods. In the first instance, yeast strains were transformed using an electroporation protocol [42], which allowed us to transform 224 strains (80.3% of the 279). For the yeast strains that could not be transformed using this method, we carried out a standard lithium acetate transformation protocol [43], which allowed us to transform an additional 50 strains (17.9% of the 279). Overall, we were able to transform 274 of the 279 strains (98.2%), a remarkably high efficiency value. Of these 274 strains, 270 of them were able to grow in YMM + Pro + Luc medium and to emit luminescence, and thus constitute the actual TOMAN-G population (Additional file 1: Table S1 and Additional file 2: Figure S1).

### Effect of domestication on TORC1 activation

Once the TOMAN-G population was obtained, we took the strains belonging to this population and phenotyped them by means of nitrogen upshift experiments (Fig. 2) to assess the activation of TORC1 in each one of them. From each luminescence curve obtained, we extracted three kinetic parameters: time at which maximum luminescence is obtained (“Time”), maximum luminescence (“Max”), and area under the luminescence curve (“AUC”), each of them for three time intervals (0–4 h, 0–12 h, and 4–12 h), giving a total of 9 different phenotypes to be compared, as previously described [35, 44]. A great phenotypic diversity was observed for the three kinetic parameters and time intervals evaluated in the entire population, corroborating the high genetic diversity that exists in the population (Additional file 1: Table S2 and Additional file 2: Figures S2-S4). Importantly, all the kinetic parameters evaluated showed continuous variation through the population (Additional file 2: Figures S2-S4), which is indicative of polygenic inheritance.

**Fig. 4:**
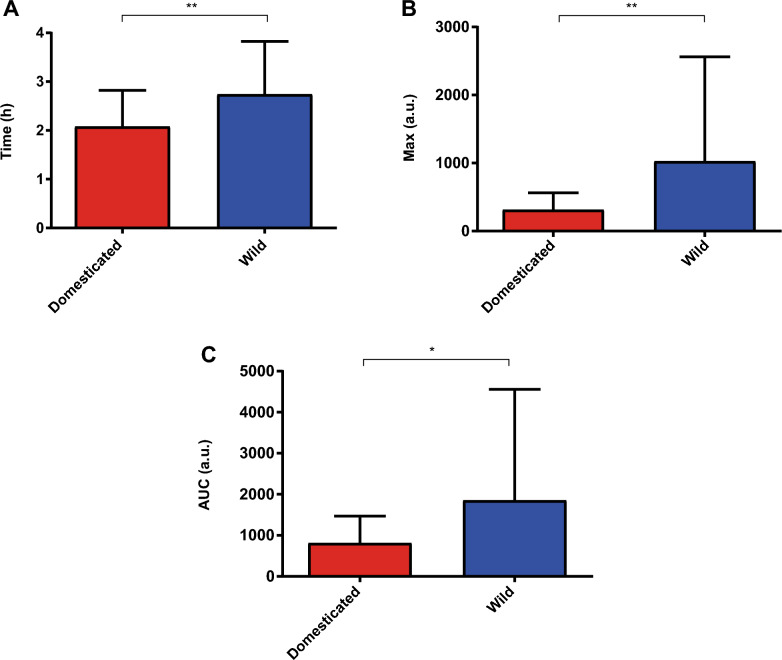
Comparison between domesticated and wild strains for the 0–4 h time interval. The kinetic parameters compared were **A** maximum luminescence time (Time), **B** maximum luminescence (Max), and **C** area under the luminescence curve (AUC). Statistical analyses correspond to two-tailed Mann Whitney tests. **: p < 0.01, *: p < 0.05

**Fig. 5:**
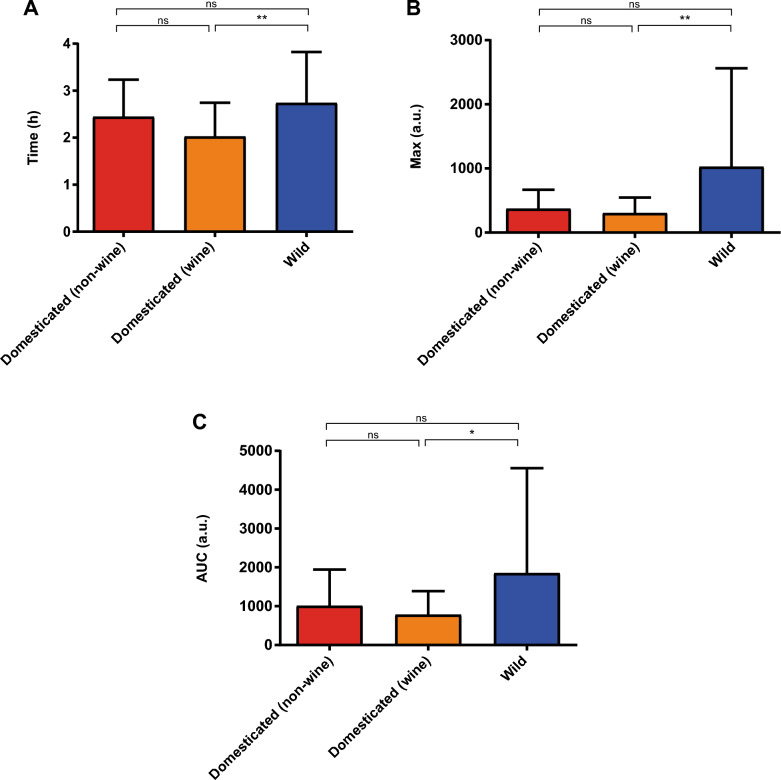
Comparison between domesticated (non-wine), domesticated (wine) and wild strains for the 0–4 h time interval. The kinetic parameters compared were **A** maximum luminescence time (Time), **B** maximum luminescence (Max), and **C** area under the luminescence curve (AUC). Statistical analyses correspond to Kruskal–Wallis tests using Dunn's multiple comparisons tests. **: p < 0.01, *: p < 0.05, ns: not significative

Once these phenotypic data were obtained, we compared the results between wild and domesticated strains, searching for an effect of domestication on the differential TORC1 activation. We especially focus on the first 4 h after the nitrogen pulse, where the first (and in many cases, only) luciferase expression maximum appears for most strains (Figs. 4, 5 and Additional file 2: Figure S2). First, we compared all domesticated strains (both wine and non-wine) versus wild strains, finding statistically significant differences for all the kinetic parameters for the first 4 h (Fig. 4), a result which is also obtained by considering the 0–12 h time interval (but not for the maximum luminescence time in the 4–12 h) (Additional file 2: Figures S5-S6). Overall, domesticated strains showed a faster but lower activation of TORC1 compared to wild strains. We also observed generally higher standard deviations for the wild strains, especially for the maximum luminescence time and the maximum luminescence parameters (Fig. 4 and Additional file 2: Figures S5-S6).

Finally, we compared the three relevant classes, *i.e.*, domesticated (non-wine), domesticated (wine) and wild. For the first 4 h, we found statistically significant differences only between domesticated (wine) and wild strains for the three kinetic parameters, although visually differences seem to be apparent between domesticated (non-wine) and wild strains (Fig. 5); a similar result was obtained for the other time intervals (Additional file 2: Figures S7-S8). Again, it was the domesticated (wine) strains that showed a faster but lower activation of TORC1 compared to the wild strains. In terms of standard deviations, we found the same trend of higher values for wild strains for the maximum luminescence time and the maximum luminescence parameters, while domesticated (non-wine) strains have in general higher values than the domesticated (wine) strains (Fig. 5 and Additional file 2: Figures S7-S8). Taken together, these results confirm phenotypic differences in TORC1 activation between wild and domesticated strains, especially when considering wine domesticated strains, with greater phenotypic variability in wild strains.

## Discussion

The study of domestication and its genetic and phenotypic effects on species is a dynamic area of interdisciplinary scientific research. Some interesting examples of phenotypic adaptions from studies in animal species include the neotonization (delayed physiological development) of cranial morphologies of domestic pigs and dogs, as well as changes in horn morphology in sheep and goats, among many others [1]. Microorganisms have also been studied for patterns of domestication, highlighting *S. cerevisiae*, possibly the most extensively studied eukaryotic microorganism [40, 41, 45–48]. Some of these studies point to domestication as one of the most dramatic events in budding yeast evolution, affecting genome dynamics [46], life cycle [40], and metabolism [45].

In the present work, we found differences in TORC1 activation between wild and domesticated yeast strains, particularly wine strains. To achieve this, we first improved a previously [28, 35] generated methodology to assess TORC1 activation (Figs. 1 and 2), which turned out to be as effective as the original but also has several practical advantages, such as facilitating confirmation of transformants and putting the *Luc* reporter gene under the control of the same *P*_*RPL26A*_ promoter for each transformed strain. Importantly, *RPL26A* was originally selected because its expression was found to be strongly activated by the TORC1 pathway [49], in addition to its minor effects on translation and the low pleiotropic effects generated by its deletion in a laboratory genetic background, according to the SGD (*Saccharomyces* Genome Database, www.yeastgenome.org) [35].

It is important to highlight the indirect nature of the TORC1 activation evaluated, in comparison to other methodologies such as the direct assessment of Sch9 [29] or Rps6 [38, 39] phosphorylation; the Sch9 kinase is the best characterized direct target of the TORC1 complex, whereas the ribosomal protein Rps6 is phosphorylated by Ypk3 (which in turn is directly phosphorylated by the TORC1 complex, such as Sch9) [29, 38, 39]. Furthermore, recent evidence points to the fact that the choice of TORC1 proxies introduces a bias in the decoding of TORC1 activity [50]. Nevertheless, the methodology used has been shown to correlate well with the results obtained when assessing Sch9 and Rps6 phosphorylation [35] and is therefore a valid way to assess TORC1 activation.

A key development of the present work is the generation of a new mapping population, the so-called TOMAN-G population, derived from the “1002 Yeast Genomes Project” population, the most complete catalogue of the existing genetic variation in the species [13]. A critical issue is what fraction of this genetic variation is still present in the TOMAN-G population compared with the original one and whether this remaining variation is useful for answering questions related to the effect of domestication on the genetic and phenotypic features of *S. cerevisiae*. It is important to note that the original “1002 Yeast Genomes Project” population already has a bias in its ecological representation towards wine strains, due to a sampling bias, with the Wine/European clade constituting more than one third of the 1011 strains (Additional file 1: Table S2 and Additional file 2: Figure S1), which had not been a limitation for using this population to study yeast genetic and phenotypic features [13, 40, 41].

The fact of considering only diploid-euploid strains slightly increased this bias towards wine strains, but a more dramatic effect was observed when considering strains capable to grow in proline as the sole nitrogen source (Fig. 3). Proline is generally considered a “non-preferred” nitrogen source, allowing slow growth rate compared to “preferred” ones (such as glutamine) [27]. Furthermore, under wine fermentation conditions, where yeast cells consume the nitrogen sources present in grape must following a similar order (“preferred” ones at the beginning of fermentation and “non-preferred” ones at later times of fermentation), proline is not consumed at all [21]. Nonetheless, proline is one of the amino acids with the highest concentration in grape musts, and in fact is the most abundant amino acid in the synthetic musts that are commonly used in laboratory experiments [51, 52], so it is possible that wine strains have better mechanisms compared to other domesticated strains (e.g., strains from the Brazilian bioethanol and African palm wine clades) to exploit this nitrogen source when no other is available. Interestingly, wild strains also seem to cope better with the situation of having proline as the only nitrogen source compared to the other domesticated strains and those of unknown origin (Fig. 3), which is in line with recent evidence pointing to a better adaptation of wild strains to nitrogen-restricted environments [47, 48].

Even considering the biases introduced by choosing diploid-euploid strains capable of growing in proline as the sole nitrogen source, the TOMAN-G population retained much of the genetic (and, therefore, phenotypic) diversity of the original “1002 Yeast Genomes Project” population. This is evidenced, on one hand, by the fact that the TOMAN-G population has at least one representative strain from 24 clades and all 3 mosaic groups of the original 26 clades and 3 mosaic groups of the “1002 Yeast Genomes Project” [13] (Additional file 1: Table S2), and, on the other hand, by the high phenotypic diversity observed when assessing TORC1 activation (Additional file 1: Table S1). This genetic diversity is key for using this population as a mapping population, which, although not the main goal of this study, could be a useful resource for future research.

Once the TOMAN-G population was generated, we were able to phenotype it for TORC1 activation and then to compare the results obtained between wild and domesticated yeast strains (Additional file 1: Table S1), in order to gain insight into the effect that domestication has had on the activation of this pleiotropic signalling pathway. When comparing wild and domesticated (wine and non-wine together) strains, we found statistically significant differences for almost all the kinetic parameters and time intervals considered (Fig. 4 and Additional file 2: Figures S5-S6), which is a strong indication of the effect of domestication on this phenotype. Moreover, higher standard deviations were obtained for the wild strains, which is consistent with a more diverse ecological origin compared to domesticated strains [13] (Additional file 1: Table S1).

When comparing wild strains with domesticated (wine) and domesticated (non-wine) strains separately, we again found statistically significant differences for almost all the kinetic parameters and time intervals considered, but only between domesticated (wine) and wild strains (Fig. 5 and Additional file 2: Figures S7-S8), corroborating that the different evolutionary trajectories of wild and domesticated (wine) strains have in fact caused differences in the activation of this pathway between them. However, although non statistically significant, visual differences seem to be evident also between domesticated (non-wine) and wild strains (and to a lesser extent, between domesticated (wine) and domesticated (non-wine) strains). A combination of a couple of reasons could explain this. First, wild and domesticated (non-wine) strains are much less in number than domesticated (wine) strains. Second, domesticated (non-wine) strains tend to have phenotypic values that are between the other two groups, although closer to domesticated (wine) strains. Third, wild and domesticated (non-wine) strains tend to have, in general, higher standard deviation values compared to domesticated (wine) strains, which is again consistent with a more diverse ecological origin [13] (Additional file 1: Table S1). Therefore, it is possible that expanding the number of strains belonging to the wild and domesticated (non-wine) classes may lead to obtaining statistically significant differences between them. Beyond that, these results are also indicative of the effect of domestication on TORC1 activation.

Overall, the domesticated (wine) strains showed faster but lower activation of TORC1 compared to both the wild and the domesticated (non-wine) strains. This is in line with previous evidence, where a wine strain showed lower TORC1 activation by glutamine than a wild strain and a sake strain [35]. Although we do not have an explanation for why domestication generated this phenomenon, it is interesting to note that other previous evidence linked this lower TORC1 activation of this wine strain with its higher fermentative capacity in a nitrogen-limited synthetic grape must [28]. Further research is needed to elucidate the evolutionary relationship between these phenotypes.

Finally, it is interesting to note that we did not only observe a high phenotypic diversity for the kinetic parameters and time intervals evaluated in the entire population (Additional file 1: Table S2 and Additional file 2: Figures S2-S4), corroborating the high genetic diversity that exists in the population, but also we found that all the kinetic parameters evaluated showed continuous variation across the population (Additional file 2: Figures S2-S4), an indication of polygenic inheritance, which is in line with previous observations on this topic [28]. These observations are indicative that the generated mapping population and the phenotypic data obtained from it could be valuable to continue underlying the genetic bases of TORC1 activation, a process which is not completely understood and for which not all participating proteins have been determined [28, 30, 53], in addition to further studying the effect of the domestication process but also considering the genomic level.

These goals can be addressed by using techniques such as QTL mapping [28], GWAS [41] and/or phylogenetic tree inferences [36, 37]. Both QTL mapping and GWAS are based on phenotype-genotype correlations, allowing the search for the genetic bases of complex traits; since the phenotypic information on TORC1 activation generated in the present work is available, and because the strains used are completely sequenced, this information could be used to search for specific single nucleotide polymorphisms (SNPs) and copy number variants (CNVs) by GWAS as previously done for other phenotypes [13, 41]. On the other hand, once this genetic information is obtained, it could be used to make phylogenetic tree inferences, which is a bioinformatic approach that could be used to reconstruct a hypothesis that explains the evolutionary relationships between a group of genes related to a specific phenotype, in this case, TORC1 activation [36, 37].

## Conclusions

In the present work, we studied the effect of domestication on TORC1 activation in *S. cerevisiae*. To achieve this goal, we improved a previously generated methodology to assess TORC1 activation, which turned out to be as effective as the original one but also presents several practical advantages for its application. Next, we generated a mapping population, the so-called TOMAN-G population, derived from the “1002 Yeast Genomes Project” population, the most complete catalogue of the genetic variation existing in yeasts. This new population retains much of the genetic diversity of the original population, which is key to using this population in mapping techniques such as GWAS. Finally, we were able to phenotype the strains belonging to the TOMAN-G population for TORC1 activation and then compare the results obtained between yeast strains with different ecological origins.

When comparing wild and domesticated (both wine and non-wine) strains, we found statistically significant differences for almost all kinetic parameters and time intervals considered, a result also obtained when considering wild and wine strains, which is indicative of the effect of domestication on TORC1 activation and a corroboration that the different evolutionary trajectories of wild and domesticated (wine) strains have in fact caused differences in the activation of this pathway between them. Furthermore, although non statistically significant, there are also differences between domesticated (non-wine) and wild strains, and to a lesser extent between domesticated (wine) and domesticated (non-wine) strains; the much lower number and higher standard deviation values of wild and domesticated (non-wine) strains compared to domesticated (wine) strains could explain this, therefore it is necessary to expand the number of strains belonging to these classes to obtain more definitive results and to better understand the role of TORC1 activation and the effect of domestication on it in different productive processes, in addition to wine fermentation.

Finally, the generated mapping population and the phenotypic data obtained from it could be valuable to continue underlying the genetic bases of TORC1 activation, a process that is still not fully understood, using techniques such as GWAS to search for specific SNPs and CNVs underlying the observed phenotypic differences between wild and domesticated strains, and also phylogenetic tree inferences to gain insight into the evolutionary relationships between these genetic variants.

## Methods

### Yeast strains

For growth assessment on YMM + Pro medium, we used 974 of the 1011 completely sequenced yeast strains belonging to the “1002 Yeast Genomes Project” [13]. For transformation with the pTOMAN-G plasmid, we considered a subset of this population, consisting only of diploid-euploid strains able to grow in a medium with proline as the sole nitrogen source (279 yeast strains in total). The final TOMAN-G population consisted of 274 strains that could be transformed. We categorized each yeast strain as “wild”, “domesticated (wine)”, “domesticated (non-wine)” or “unknown”, following previous criteria [40, 41]. All yeast strains used are listed in Additional file 1: Table S1.

### Plasmid construction and transformation

#### pTOMAN-G plasmid

The constructed recombinant plasmid is shown in Fig. 1. This plasmid was designed *in silico* using the molecular biology software Benchling (https://www.benchling.com/) and was assembled in vivo to contain a transcriptional fusion controlled by the *RPL26A* promoter of the BY4741 yeast strain (*P*_*RPL26A*_), using as a backbone the pRS316 plasmid, a centromeric yeast shuttle vector. For this purpose, a 631 bp fragment of the *P*_*RPL26A*_ [35] was fused to the luciferase reporter gene (*Luc*). The *Luc* gene encodes a destabilized version of the firefly luciferase gene, which contains an ARE sequence for mRNA destabilization, and a PEST sequence for proteasome-mediated degradation of the luciferase protein [54]. In addition, a hygromycin resistance cassette (*HphMx*) was added downstream the *Luc* sequence as a selectable marker. Each fragment of the genetic construct was amplified by PCR using the Phusion flash high-fidelity master mix (Thermo Fisher Scientific, USA). The primers used in the PCR reactions contained 20 nt for amplification of each fragment plus 30 nt overhangs with adjacent PCR fragments. Then, the PCR products were co-transformed and cloned into the centromeric plasmid pRS316 using Yeast Recombinational Cloning (YRC) [55]. Afterwards, the assembled plasmid was transferred to *Escherichia coli* and confirmed by standard colony PCR. Finally, the genetic construct was confirmed by Sanger sequencing (Macrogen Inc., Republic of Korea).

#### Transformation protocols

In the first instance, yeast strains were transformed using a standard electroporation protocol [42]. For those strains that could not be transformed using this method, we employed a standard lithium acetate transformation protocol [43].

### Growth assessment

The growth capacity of strains from the “1002 Yeast Genomes Project” population on a medium with proline as the sole nitrogen source was assessed by monitoring the optical density at 600 nm (OD_600_) of cells under microculture conditions, as previously described [41]. Briefly, yeast strains were grown at 30 ºC in 96-well plates containing 200 µL of yeast minimal medium (YMM) (20 g/L glucose and 1.7 g/L yeast nitrogen base without amino acids and without ammonium sulphate) supplemented with proline (0.5 mg/mL) (YMM + Pro). OD_600_ was measured using 30 min intervals on a Tecan Sunrise microplate reader (Tecan, Germany) for up to 46 h. All microculture experiments were conducted in two independent biological replicates.

### TORC1 activation assessment

#### Nitrogen upshift experiments

TORC1 activation was assessed in strains carrying the pTOMAN-G plasmid by simultaneously monitoring the OD_600_ and luminescence of cells under microculture conditions, as previously described [35, 44]. An overview of this method is shown in Fig. 2. Briefly, we performed nitrogen upshift experiments, where the strains were grown at 30 ºC in 96-well plates containing 300 µL of YMM + Pro medium, supplemented with luciferin (1 mM) and until OD_600_ ~ 0.8. Then, 10 µL of glutamine (15 mg/mL; 0.5 mg/mL final concentration) were added [35]. Luminescence was measured using 10 min intervals on a Synergy HTX microplate reader (Biotek, USA) for up to 12 h. From each luminescence curve, we extracted three kinetic parameters (time at which maximum luminescence is obtained (“Time”), maximum luminescence (“Max”) and area under the curve of luminescence (“AUC”)) for three time intervals (0–4 h, 0–12 h, and 4–12 h), using Graph Pad Prism 7.04 software. All microculture experiments were conducted in three independent biological replicates.

### Statistical analysis

Statistical analysis of the kinetic parameters between different classes was also performed using Graph Pad Prism 7.04 software. For comparisons between domesticated and wild strains, statistical analysis consisted of two-tailed Mann Whitney tests, while for comparisons between domesticated (non-wine), domesticated (wine), and wild strains, statistical analysis consisted of Kruskal–Wallis tests using Dunn's multiple comparisons tests.

## Supplementary Information

Supplementary Material 1. Table S1: Phenotypic, genomic and ecological data of the TOMAN-G population. Table S2: Number of strains by clade in the TOMAN-G population.

Supplementary Material 2. Figure S1: Class composition of the different populations under study. Figure S2: TORC1 activation phenotypes of the TOMAN-G population for the 0–4 h time interval. Figure S3: TORC1 activation phenotypes of the TOMAN-G population for the 0–12 h time interval. Figure S4: TORC1 activation phenotypes of the TOMAN-G population for the 4–12 h time interval. Figure S5: Comparison between domesticated and wild strains for the 0–12 h time interval. Figure S6: Comparison between domesticated and wild strains for the 4–12 h time interval. Figure S7: Comparison between domesticated (non-wine), domesticated (wine) and wild strains for the 0–12 h time interval. Figure S8: Comparison between domesticated (non-wine), domesticated (wine) and wild strains for the 4–12 h time interval.

## Data Availability

The datasets supporting the conclusions of this article are included within the article (and its additional files).

## Abbreviations

ANOVA: Analysis of variance
ARS: Autonomously replicating sequence
AUC: Area under the curve
CNV: Copy number variant
GAAC: General amino acid control
GWAS: Genome-wide association study
NCR: Nitrogen catabolic repression
OD_600_: Optical density at 600 nm
ORF: Open reading frame
QTL: Quantitative trait locus
RTG: Retrograde signalling pathway
SGD: *Saccharomyces* Genome Database
SNP: Single nucleotide polymorphism
SPS: Ssy1-Ptr3-Ssy5 system
TOMAN-G: TORC1 activation Mapping of New variants by GWAS
YMM: Yeast minimal medium
YRC: Yeast Recombinational Cloning
