# Supplementary material for: Phenotyping of a new yeast mapping population reveals differences in the activation of the TORC1 signalling pathway between wild and domesticated yeast strains
Source: Biol Res. 2024 Nov 7;57:82. doi: 10.1186/s40659-024-00563-5 (PMC11545388; doi:10.1186/s40659-024-00563-5)
Supplement: Supplementary file 2 — Supplementary Material 2. Figure S1: Class composition of the different populations under study. Figure S2: TORC1 activation phenotypes of the TOMAN-G population for the 0–4 h time interval. Figure S3: TORC1 activation phenotypes of the TOMAN-G population for the 0–12 h time interval. Figure S4: TORC1 activation phenotypes of the TOMAN-G population for the 4–12 h time interval. Figure S5: Comparison between domesticated and wild strains for the 0–12 h time interval. Figure S6: Comparison between domesticated and wild strains for the 4–12 h time interval. Figure S7: Comparison between domesticated (non-wine), domesticated (wine) and wild strains for the 0–12 h time interval. Figure S8: Comparison between domesticated (non-wine), domesticated (wine) and wild strains for the 4–12 h time interval. [file 40659_2024_563_MOESM2_ESM.pdf]

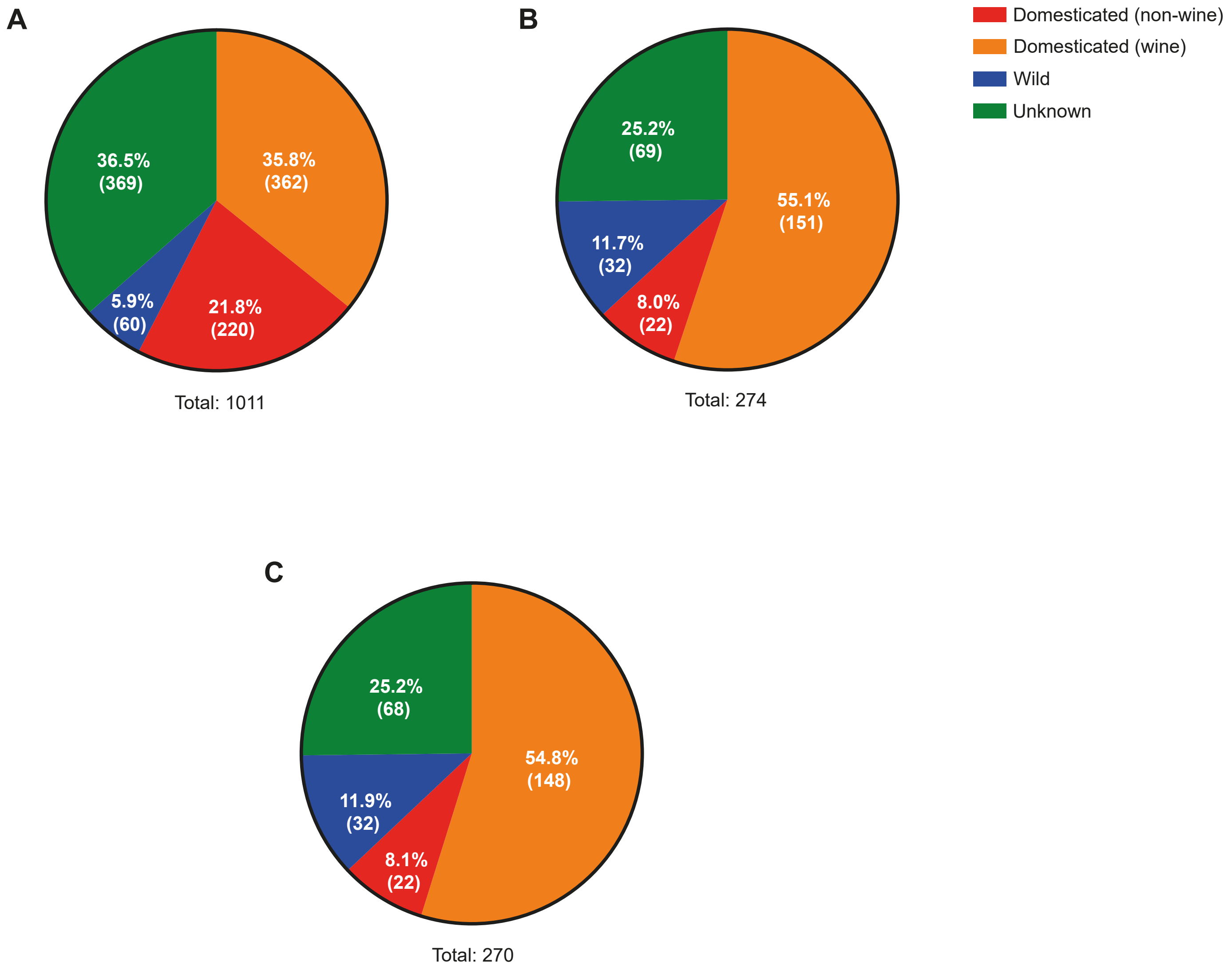

Figure S1. Class composition of the different populations under study. The yeast strains in each group are those (A) belonging to the “1002 Yeast Genomes Project” population, (B) transformed with the pTOMAN-G plasmid, and (C) phenotyped for TORC1 activation.

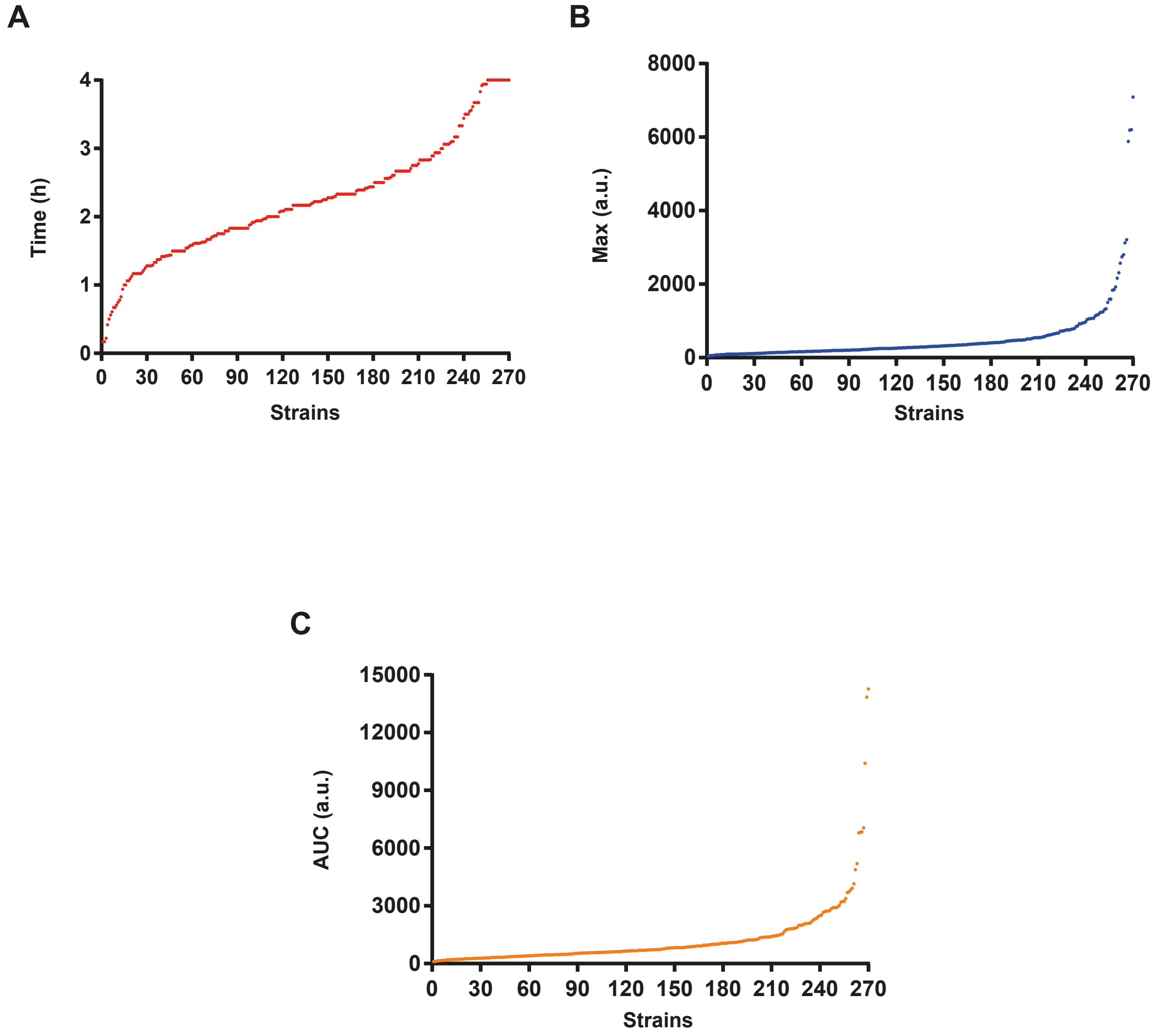

Figure S2. TORC1 activation phenotypes of the TOMAN-G population for the 0-4 h time interval. Segregants were ordered from lowest to highest phenotypic value obtained for the kinetic parameters of (A) maximum luminescence time (Time), (B) maximum luminescence (Max), and (C) area under the luminescence curve (AUC).

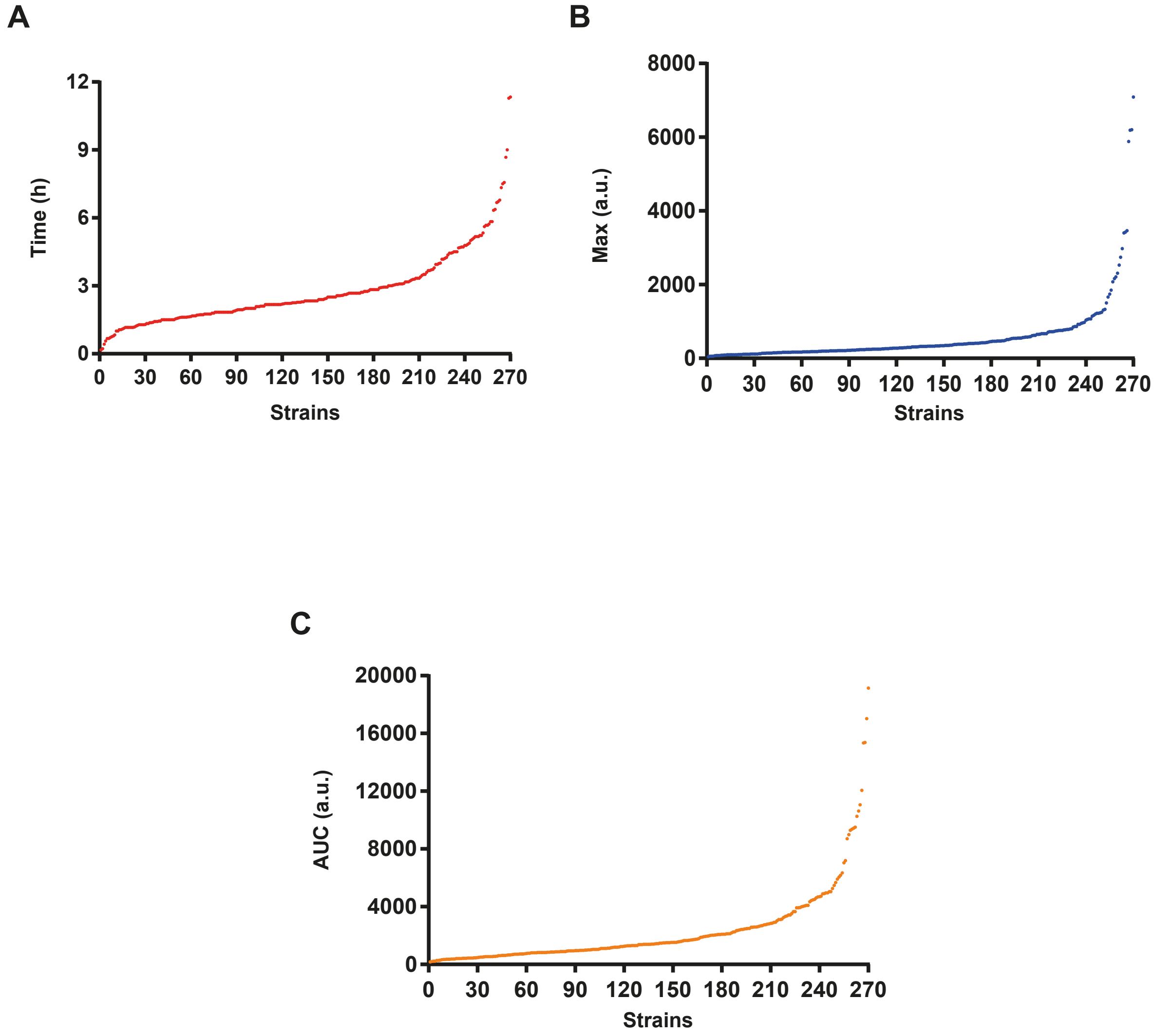

Figure S3. TORC1 activation phenotypes of the TOMAN-G population for the 0-12 h time interval. Segregants were ordered from lowest to highest phenotypic value obtained for the kinetic parameters of (A) maximum luminescence time (Time), (B) maximum luminescence (Max), and (C) area under the luminescence curve (AUC).

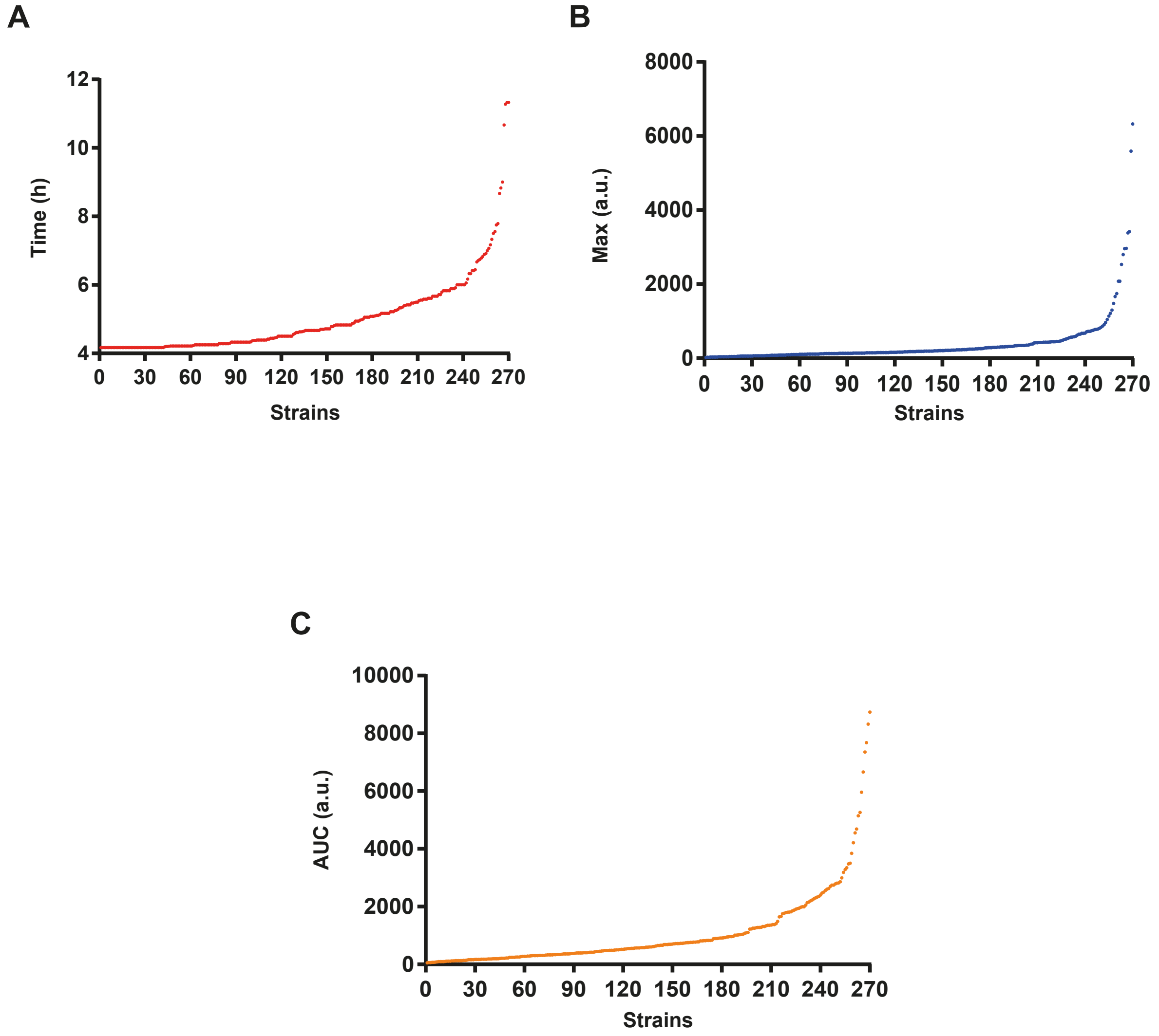

Figure S4. TORC1 activation phenotypes of the TOMAN-G population for the 4-12 h time interval. Segregants were ordered from lowest to highest phenotypic value obtained for the kinetic parameters of (A) maximum luminescence time (Time), (B) maximum luminescence (Max), and (C) area under the luminescence curve (AUC).

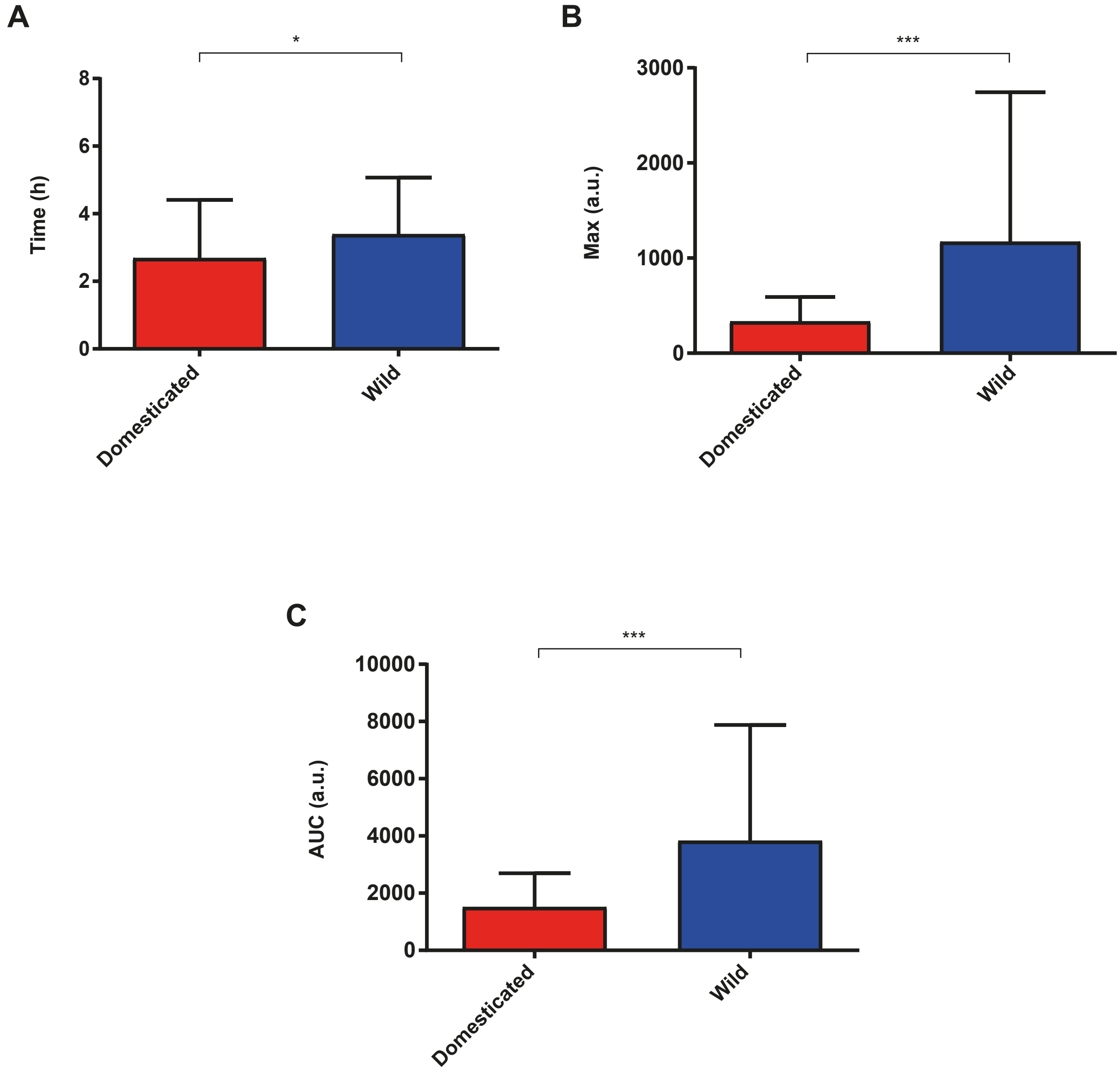

Figure S5. Comparison between domesticated and wild strains for the 0-12 h time interval. The kinetic parameters compared were (A) maximum luminescence time (Time), (B) maximum luminescence (Max), and (C) area under the luminescence curve (AUC). Statistical analyses correspond ttwo-tailed Mann Whitney tests. ***: $p<0.001$, *: $p<0.05$.

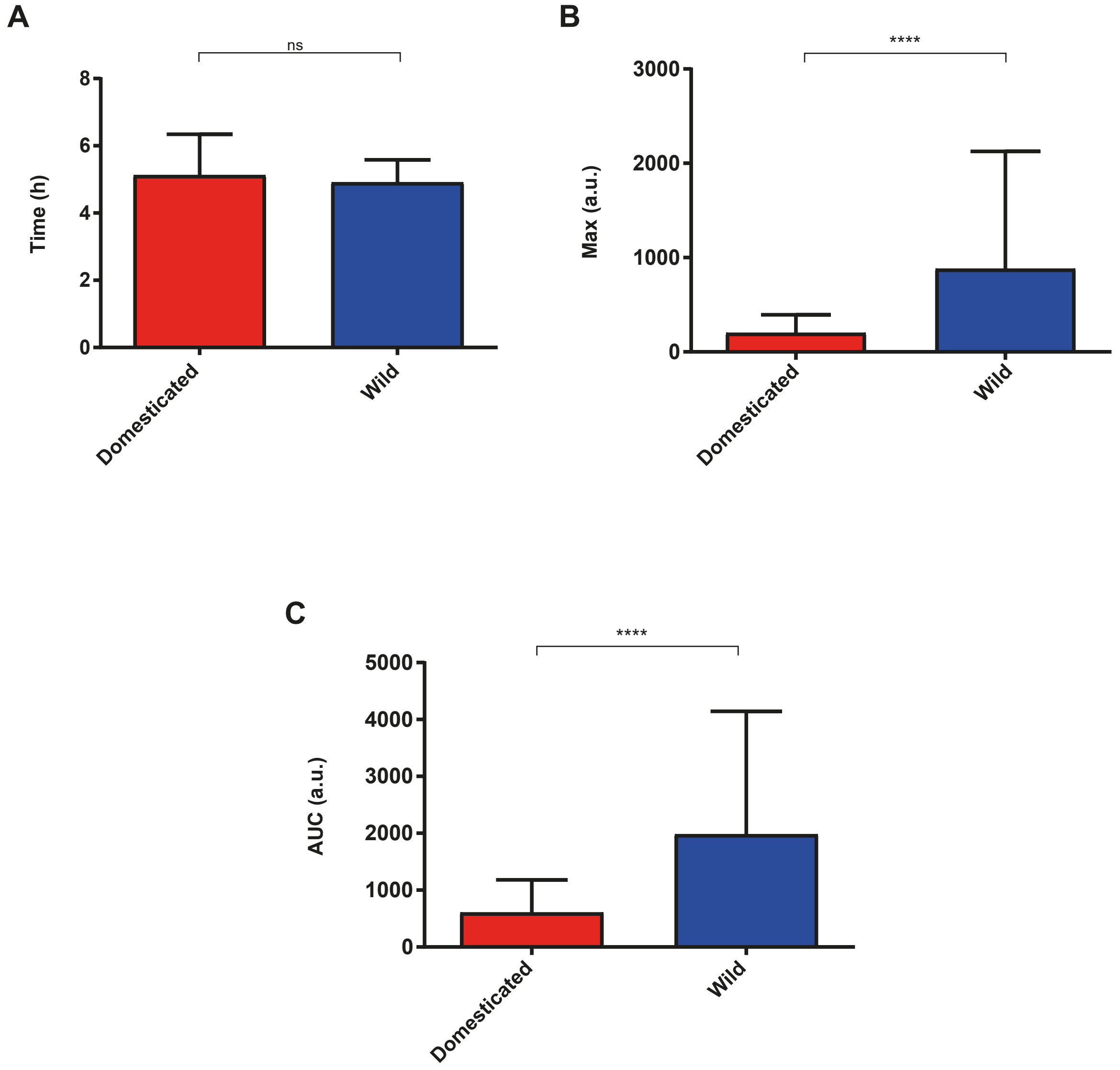

Figure S6. Comparison between domesticated and wild strains for the 4-12 h time interval. The kinetic parameters compared were (A) maximum luminescence time (Time), (B) maximum luminescence (Max), and (C) area under the luminescence curve (AUC). Statistical analyses correspond ttwo-tailed Mann Whitney tests. ****: $p<0.0001$, ns: not significative.

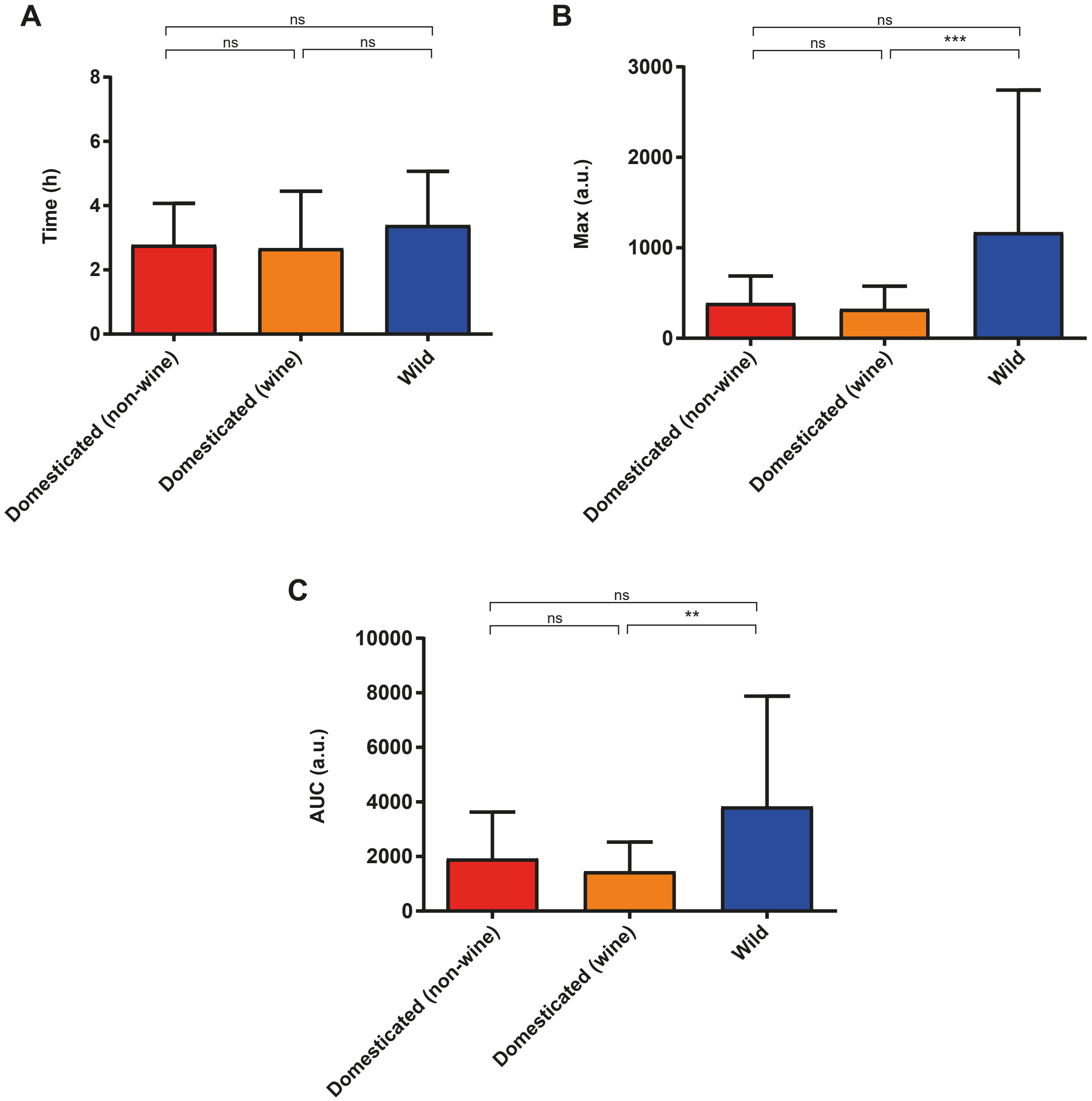

Figure S7. Comparison between domesticated (non-wine), domesticated (wine) and wild strains for the 0-12 h time interval. The kinetic parameters compared were (A) maximum luminescence time (Time), (B) maximum luminescence (Max), and (C) area under the luminescence curve (AUC). Statistical analyses correspond to Kruskal-Wallis tests using Dunn's multiple comparisons tests. ***: $p<0.001$, **: $p<0.01$, ns: not significative.

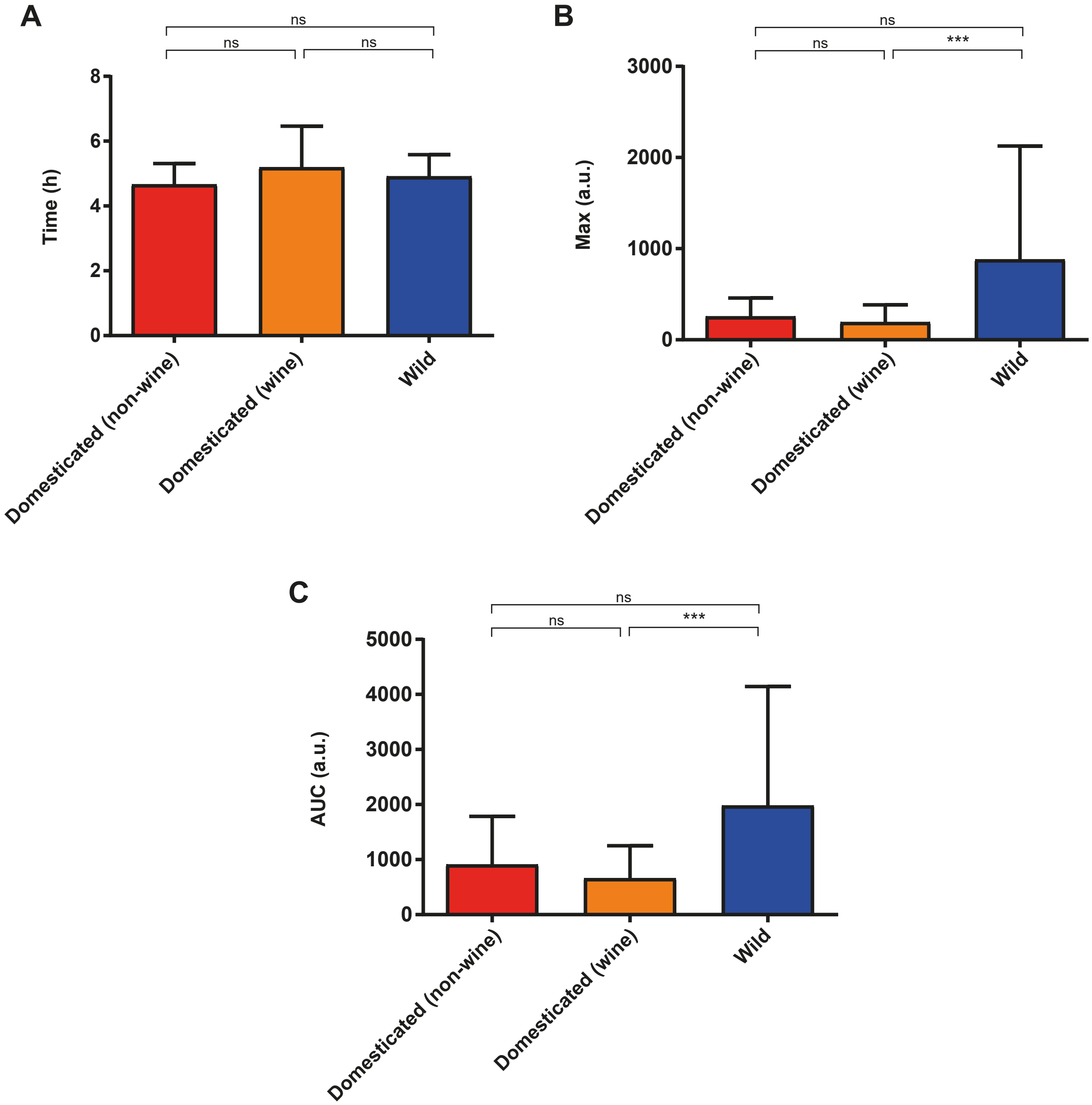

Figure S8. Comparison between domesticated (non-wine), domesticated (wine) and wild strains for the 4-12 h time interval. The kinetic parameters compared were (A) maximum luminescence time (Time), (B) maximum luminescence (Max), and (C) area under the luminescence curve (AUC). Statistical analyses correspond to Kruskal-Wallis tests using Dunn's multiple comparisons tests. ***: $p<0.001$, ns: not significative.
